# Reducing Low Anterior Resection Syndrome After Low Rectal Cancer Surgery Using an Integrated Intra-Anal Balloon Training and Reminder Device: Feasibility Nonrandomized Controlled Trial

**DOI:** 10.2196/91332

**Published:** 2026-05-08

**Authors:** Qing Zhang, Yanjun Wang, Meiling Wang, Haiyan Hu, Quan Wang, Yuchen Guo, Jianan Sun, Xuan Sun

**Affiliations:** 1 Department of Gastrocolorectal Surgery, General Surgery Center, The First Hospital of Jilin University, No. 1 Xinmin Street, Chaoyang District, Changchun, Jilin, 130012, China, 86 15804303726

**Keywords:** pelvic floor muscle training, rectal cancer surgery, low anterior resection syndrome, quality of life, biofeedback device

## Abstract

**Background:**

Low anterior resection syndrome (LARS) is a common postoperative complication in patients with low rectal cancer, presenting with a spectrum of bowel dysfunction symptoms, including urgency, incontinence, evacuation disorders, and changes in stool frequency. Pelvic floor muscle training (PFMT) can alleviate LARS, but its effectiveness may be limited by poor accuracy of technique and low adherence during home-based training due to a lack of real-time feedback and monitoring devices.

**Objective:**

This study aimed to evaluate the effects of a novel integrated balloon biofeedback device for home-based PFMT on accuracy of technique, adherence, quality of life, and LARS reduction in patients after sphincter-preserving surgery for low rectal cancer.

**Methods:**

A nonrandomized controlled trial was conducted among 164 patients with low rectal cancer who underwent temporary ileostomy without neoadjuvant therapy. Participants were assigned by surgical date to an intervention group (n=82) using an adjustable-pressure balloon device with real-time waveform feedback via a mobile app or a control group (n=82) performing PFMT without equipment. PFMT was initiated within 72 hours after temporary ileostomy and continued throughout the stoma period until 1 month after ileostomy reversal. Outcomes including PFMT accuracy of technique, adherence, LARS score and incidence, and quality of life (European Organisation for Research and Treatment of Cancer Quality of Life Questionnaire Core 30) were assessed 1 month after stoma reversal.

**Results:**

At baseline, major LARS was present in 47.6% (39/82) of patients in the intervention group and 62.2% (51/82) of patients in the control group. Compared with the control group, the intervention group showed significantly higher PFMT training accuracy and adherence (*P*<.001 in both cases). LARS scores were significantly lower in the intervention group (median 16.00, IQR 11.00-29.00 vs 32.00, IQR 13.75-36.00), with a markedly reduced proportion of major LARS (17/82, 20.7% vs 45/82, 54.9%; *P*<.001). Global health/quality of life scores were significantly higher in the intervention group (*P*<.001).

**Conclusions:**

The integrated balloon biofeedback device improved the accuracy of technique and adherence to home-based PFMT, reduced the incidence and severity of LARS, and enhanced quality of life in patients after low rectal cancer surgery. These findings support further development and clinical implementation of the device. However, the nonrandomized, time-sequenced study design and baseline differences between groups may limit causal interpretation of the results, and randomized controlled trials with longer follow-ups are needed to confirm long-term efficacy.

## Introduction

Rectal cancer is one of the most common malignancies [1], with low rectal cancer accounting for approximately 75% of rectal cancers. In recent years, with the continuous improvement in surgical techniques, preserving the anal sphincter to avoid permanent ostomy has become the primary choice for more patients with low rectal cancer [2]. However, the reality is that approximately 90% of patients face a series of intestinal dysfunctions after surgery, known as low anterior resection syndrome (LARS), characterized by unpredictable features such as frequent bowel movements, urgency, and fecal incontinence [3,4], which seriously affect patients’ social and quality of life [5]. Currently, the incidence of major LARS is close to 50%, with half of the patients experiencing symptoms for up to 15 years [6].

Pelvic floor muscle training (PFMT), as one of the important interventions for postoperative pelvic floor rehabilitation in patients with rectal cancer, works through bidirectional regulation of pelvic floor muscle function. PFMT works by increasing the voluntary contractile capacity of the external anal sphincter and levator ani muscles. PFMT improves fecal incontinence symptoms in patients with LARS; conversely, in cases of pelvic floor hypertonicity or spasm, PFMT alleviates symptoms by relaxing the pelvic floor muscles, effectively reducing urgency and spasmodic bowel symptoms [7]. By restoring mechanical balance to the pelvic floor muscles and optimizing anatomical structures such as the anorectal angle, PFMT improves postoperative LARS symptoms and quality of life in patients with rectal cancer [8-10]. PFMT is recommended by many scholars for its advantages of being noninvasive, requiring no additional equipment, being low cost, and allowing patients to exercise at home [9,11]. However, studies have found that PFMT adherence is relatively low [12]. Moreover, research indicates that many individuals struggle to correctly perform pelvic floor muscle contractions. In a study on postmenopausal women, 32% were unable to correctly perform their first pelvic floor muscle contraction without guidance [13]. Similarly, a study of 82 women found that, although 98.8% believed that they could voluntarily contract their pelvic floor muscles, only 33% accurately estimated their contraction when assessed by a physiotherapist [14]. Even in women with no or mild pelvic floor disorders, 14% to 31% could not correctly contract on first attempt, particularly those with pelvic organ prolapse [15]. For patients who have undergone low anterior resection—where pelvic floor anatomy and innervation may be compromised—these challenges are likely even more pronounced. The lack of effective and objective real-time feedback may be a key bottleneck restricting the efficacy of home-based PFMT.

To address this bottleneck, we have developed a novel home-use integrated pelvic floor balloon biofeedback training device. Its design is based on a clear physiological rationale: active contraction of the pelvic floor muscles increases anal canal pressure, which is a key mechanism for maintaining fecal continence [16]. In addition to improving voluntary contraction during training, PFMT may induce muscle hypertrophy, increasing the bulk of the muscles around the anal canal and providing greater structural support even at rest [17]. The device places a balloon sensor in the anorectal ring region to directly capture the specific pressure signals generated through pelvic floor muscle contraction. Through real-time pressure-waveform conversion and intelligent algorithm analysis, it provides users with immediate, objective feedback on the accuracy of technique of their contractions. This process establishes a complete biofeedback loop, and research has confirmed that transforming intrinsic physiological signals into intuitive visual cues can effectively guide patients in identifying and regulating pelvic floor muscle function [18]. On the basis of this, we hypothesized that this device, through targeted biomechanical signal-based precise biofeedback, could guide patients in performing correct home-based PFMT; improve the accuracy of technique of movements and training adherence; and, ultimately, enhance the neuromuscular control capacity of the pelvic floor muscles to improve postoperative bowel function and reduce the incidence and severity of LARS. This study aimed to evaluate the effects of this device on PFMT outcomes and the occurrence and progression of LARS at 1 month postoperatively in patients who have undergone sphincter-preserving surgery for low rectal cancer.

## Methods

### Participants

Patients with rectal cancer admitted to the Department of Gastrointestinal Surgery at the First Hospital of Jilin University from October 2022 to March 2023 were enrolled as the control group, whereas patients admitted from April 2023 to September 2023 were enrolled as the intervention group. Inclusion criteria were (1) age of 18 years or above, (2) pathological diagnosis of low rectal cancer (tumor edge located ≤5 cm from the dentate line) and confirmed through pelvic magnetic resonance imaging; (3) having undergone temporary ileostomy at the time of rectal surgery, followed by ileostomy reversal surgery 3 months postoperatively; (4) consciousness and ability to communicate without barriers; and (5) independence in daily living and ability to understand and consent to participate in the study. Exclusion criteria were (1) distant metastasis; (2) concurrent tumors in other organs; (3) preoperative presence of severe conditions such as severe hemorrhoids, anal fissures, anal fistulas, rectal prolapse, irritable bowel syndrome, Crohn disease, and ulcerative colitis; (4) having received preoperative neoadjuvant therapy; (5) unfeasibility of temporary ileostomy reversal; (6) postoperative complications related to anastomotic leakage; and (7) voluntary withdrawal from treatment, death during the study, or loss to follow-up. The eligibility criteria above were applied uniformly to both the control and intervention groups prior to allocation.

### Intervention Methods

All PFMT guidance and follow-up in both groups were provided by specialized pelvic floor rehabilitation nurses with postgraduate qualifications in pelvic floor rehabilitation and a mean of 13 (SD 8) years of clinical experience in this field.

#### Control Group

The control group received routine PFMT guidance and follow-up. First, during the preoperative stage of temporary ileostomy surgery, specialized pelvic floor rehabilitation nurses explained the potential hazards of LARS to patients and introduced PFMT methods; PFMT was initiated within 72 hours after temporary ileostomy and continued throughout the stoma period until 1 month after ileostomy reversal. Patients also received lifestyle advice, including regarding adequate fluid intake, a high-fiber diet to prevent constipation, and avoidance of straining during bowel movements. Second, personalized PFMT guidance was provided to patients through educational videos, manuals, and classes. Patients were instructed to contract the pelvic floor muscles as if “stopping the passage of stool” or “holding in gas,” lifting the muscles upward and inward. Each contraction was held for 3 to 10 seconds (initially based on patients’ self-reported ability and subsequently adjusted based on the 1-month biofeedback assessment), followed by complete relaxation for an equal period. Patients were advised to avoid holding their breath or contracting abdominal muscles and were registered for an electronic training diary. Third, during the period from discharge to 1 month after temporary ileostomy reversal surgery, follow-up nurses sent training voice messages and videos to patients daily through individual WeChat groups, requiring patients to complete 30 minutes of PFMT daily, which could be done multiple times to achieve the target volume. Follow-up nurses were responsible for supervising patients in filling out the electronic training diary. Motivation strategies included daily reminders and goal setting (completing 30 minutes daily). Fourth, during the outpatient review 1 month postoperatively, specialized pelvic floor rehabilitation nurses assessed patients’ pelvic floor muscle function using a biofeedback device (SA9800; Thought Technology Ltd; see the section PFMT Accuracy of Technique for a detailed device description) to determine each patient’s baseline maximum voluntary contraction, which informed the starting level for ongoing training; provided targeted PFMT re-education based on the assessment results; and emphasized once again that the training duration was until 1 month after temporary ileostomy reversal surgery. Patients were instructed to progress their training by (1) increasing hold duration from 3 to 10 seconds as tolerated, (2) increasing repetitions, (3) changing positions (lying, sitting, and standing), and (4) incorporating functional activities (eg, contracting during coughing). Progression was based on perceived ability and successful completion of the current level. Fifth, 1 month after temporary ileostomy reversal surgery, specialized pelvic floor rehabilitation nurses assessed patients’ training adherence, accuracy of technique, occurrence of LARS, and quality of life in the outpatient setting. Sixth, based on the assessment results, further training and treatment recommendations were provided to patients.

#### Intervention Group

The intervention group received the following additional care beyond the control protocol: pelvic floor rehabilitation specialist nurses provided 2 guidance sessions. The first session was conducted preoperatively, introducing the concept and importance of PFMT. The second session occurred during hospitalization after temporary ileostomy surgery on home-based pelvic floor intra-anal balloon training and the operation of the integrated reminder device. Before initiation of balloon-assisted training, participants in the intervention group underwent a 1-month postoperative digital rectal examination (DRE) as a safety prerequisite for device use. This assessment was not part of the baseline eligibility criteria.

The intervention was implemented in 2 distinct phases. Phase 1 was early PFMT (noninvasive). Patients commenced standard, noninvasive pelvic floor muscle contractions and relaxation exercises (ie, Kegel exercises) within 72 hours after temporary ileostomy surgery. This phase aimed to prevent muscle disuse atrophy and promote neuromuscular re-education without any device insertion or mechanical contact with the nascent anastomosis. Phase 2 was intra-anal balloon training (device assisted). The initiation of this phase was strictly contingent upon passing a safety assessment conducted during the 1-month postoperative outpatient follow-up. Physicians assessed anastomotic healing status through (1) DRE to confirm anastomotic patency (defined as the passage of the distal phalanx of the index finger); (2) endoscopic evaluation to exclude mucosal erythema, ulceration, or fibrotic stenosis; and (3) pain assessment using a visual analog scale (score of <4) induced via contact with the anastomosis during DRE. This safety assessment was performed only for patients in the intervention group to determine their eligibility to begin balloon-assisted training and did not influence participant allocation, which had already been completed prior to the assessment. Only patients demonstrating satisfactory healing based on all the criteria above received reinforced guidance on home-based pelvic floor intra-anal balloon training and device use, officially initiating the intra-anal balloon training regimen. Patients were instructed to progress their training by (1) increasing hold duration from 3 to 10 seconds as tolerated, (2) increasing repetitions, (3) changing positions (lying to sitting to standing), and (4) incorporating functional activities (eg, contracting during coughing). Progression was based on perceived ability and successful completion of the current level.

**Figure 1.**
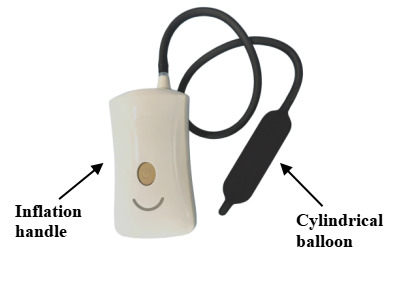
Intra-anal balloon training device.

**Figure 2.**
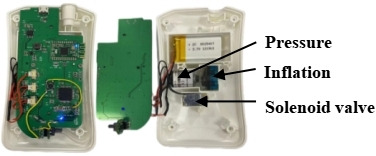
Internal structure of the inflation handle.

**Figure 3.**
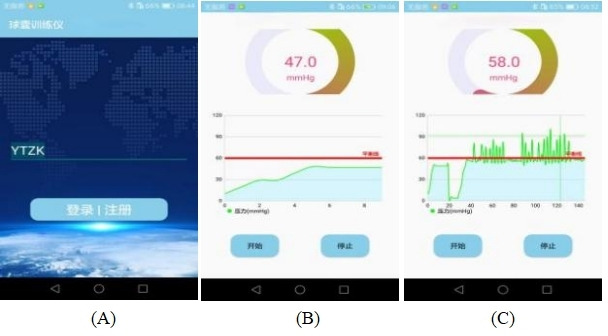
(A) App log-in screen, (B) inflation interface, and (C) training interface.

The development of the intra-anal balloon training and reminder integrated device was based on 2 previously approved patents: one for a PFMT device inserted anally (patent ZL 2017 2 1594601.5) and another for a PFMT device displaying pressure values (patent ZL 2021 2 0383459.X). The device consists of 2 main components: an adjustable-pressure intra-anal balloon training device, with hardware including a cylindrical balloon and an inflation handle (as shown in Figure 1). The cylindrical balloon is made of medical-grade silicone material, has passed biocompatibility testing, and is designed to be soft and comfortable and conform to the intestinal wall. It is adaptable and retractable, with a diameter of 2.5 cm and a cylindrical length of 8 cm. Health care providers set the inflation threshold not exceeding 50 mm Hg based on the patient’s initial sensation threshold determined through anorectal manometry. The inflation handle contains an inflation pump, solenoid valve, and pressure sensor (as shown in Figure 2). The device uses pressure exchange and solenoid valve control principles to monitor pressure, control inflation, and compress the balloon. The second component of the device is the companion app, which connects to the intra-anal balloon training device via Bluetooth. Upon log-in, the app automatically switches to the inflation interface, followed by the training interface after inflation, displaying real-time pressure waveforms during training (as shown in Figure 3). It should be emphasized that the pressure signals recorded by this device possess a clear physiological origin and specificity. When the balloon is positioned in the anorectal ring region, the perceived pressure changes mainly originate from the active contraction of the pelvic floor muscle complex, particularly the direct radial compression exerted by the external anal sphincter and the synergistic upward traction provided by the puborectalis muscle. A correct and coordinated pelvic floor muscle contraction is manifested on the waveform as a pressure curve characterized by a gentle rise, stable maintenance, and gradual decline, reflecting effective force generation by the pelvic floor muscles themselves. Conversely, if the waveform exhibits a sharp rise and fall, lacks a stable plateau, or displays jagged fluctuations, it suggests possible compensatory contractions such as those of the abdominal muscles leading to intra-abdominal pressure transmission, which represents an erroneous pattern to be avoided. Our hypothesis is that this device not only provides real-time pressure monitoring but also, through the recognition and feedback of waveform characteristics, guides patients to achieve precise, pelvic floor muscle–dominated contractions that are independent of abdominal pressure interference during training, thereby enhancing neuromuscular coordination between pelvic floor muscle contraction and abdominal pressure regulation.

The specific instructions for using the device were as follows. Initially, the specialized pelvic floor rehabilitation nurse adjusted the training inflation threshold for each patient based on preoperative anorectal manometry results obtained using a clinical anorectal manometry system (Hefei Aoyuan Technology Development Co, Ltd; model ZGJ-D3), and guided the patient to install the training software and enter the training interface. The patient was then placed in the left lateral position. After lubricating the balloon with liquid paraffin, it was gently and slowly inserted through the anus until the end of the cylindrical balloon was positioned within the anal canal region (approximately 1-2 cm from the anal verge). The patient then assumed a supine position in preparation for training. To start the training, the patient clicked the Inflate button, and the balloon inflated to the set pressure threshold. After inflation stopped, clicking Start prompted the patient to perform pelvic floor muscle contractions and relaxations following voice prompts. During the Train and Hold process, the patient contracted the anus forcefully for 10 seconds, mimicking the act of holding in a bowel movement. The pressure exerted by the wall of the anal canal on the balloon during this contraction was monitored, and when the pressure exceeded the inflation threshold, the device emitted a beep to indicate successful training. Throughout the training, the pressure sensor provided feedback to the mobile app, displaying real-time pressure values and waveform trends during the contraction and relaxation phases. The patient could adjust the training intensity based on the numerical and waveform prompts. After the voice prompt indicated “training complete,” the balloon was deflated and then slowly withdrawn from the anus. Additionally, it is important to note that the intra-anal balloon training and reminder integrated device used in this study is a self-designed prototype. This device is not a commercially available product and has not been approved by the Food and Drug Administration for any purpose. Its use in this study was investigational and specifically designed to evaluate its potential in guiding and monitoring PFMT for patients with low rectal cancer.

The research team monitored protocol adherence through regular review of electronic training diaries and WeChat communication records. No major protocol deviations were reported.

### Evaluation Indicators

#### PFMT Accuracy of Technique

At the 1-month follow-up after temporary ileostomy reversal surgery, the accuracy of technique of the patients’ training was assessed using a biofeedback device (SA9800 by Thought Technology Ltd). The biofeedback device was connected to the patients’ abdominal electrodes to collect electromyographic signals and display them in real time on a computer screen. The waveform moved upward with abdominal exertion and downward with relaxation. If the patient exerted excessive abdominal force beyond the threshold, a blue threshold line appeared on the waveform. During the 10-minute training session, the total time the blue threshold line was present represented incorrect training time, whereas the rest indicated correct training time (accuracy of technique = correct training time/10 minutes × 100%).

#### Adherence to PFMT

The daily training time of patients in both groups was obtained through self-recorded electronic training diaries. The daily training target duration was 30 minutes (daily training adherence = total training time on a given day/30 minutes × 100%). If the training duration exceeded 30 minutes, adherence was set at 100%. The highest adherence value was 100%. The average adherence during the 3-month training period following temporary ileostomy was calculated based on daily adherence records.

#### LARS Score

The LARS score developed by Emmertsen and Laurberg [19] in 2012 was used for evaluation. This score is specifically designed to assess bowel function in patients after low anterior resection surgery, identifying the occurrence and severity of LARS. The LARS score consists of 5 items: incontinence to gas, incontinence to liquid stool, bowel frequency, repeated defecation within 1 hour (clustering of stool), and fecal urgency. The total score is 42, with higher scores indicating worse bowel function. LARS is classified as major (score of ≥30), minor (score of 21-29), and no LARS (score of ≤20).

#### Quality of Life

The European Organisation for Research and Treatment of Cancer Quality of Life Questionnaire Core 30 [20] was used to assess the quality of life of the 2 groups of patients at 1 month postoperatively. This questionnaire includes 5 subscales for physical, role, cognitive, emotional, and social functioning; 3 symptom subscales for pain, fatigue, and nausea or vomiting; 6 single-item scales for dyspnea, appetite loss, insomnia, constipation, financial difficulties, and diarrhea; and 1 overall quality of life scale. Each item is scored from 0 to 100, with higher scores indicating a higher level of quality of life.

#### Adverse Events

Adverse events were defined as any device-related complications, including but not limited to anal pain, bleeding, mucosal injury, or infection. Patients in both groups were instructed to report any discomfort, pain, or unusual symptoms during the training period at each follow-up contact (including daily WeChat follow-ups and outpatient visits). All reported events were recorded and assessed by the research team for severity and causality.

### Sample Size Calculation

This study was a nonrandomized controlled trial. The intervention group underwent PFMT using the intra-anal balloon training and reminder integrated device, whereas the control group received routine PFMT. The primary outcome measure was the LARS incidence rate at 1 month postoperatively. On the basis of prior experimental results, the LARS incidence rate in the control group was approximately 75%. We expected the LARS incidence rate in the intervention group to decrease to 50%. With a 2-sided α of .05 and a power of 90%, using the PASS software (version 15; NCSS LLC), the calculated sample size was 74 patients for the treatment group and 74 patients for the control group. Considering a 10% attrition and refusal rate, the final required sample size was at least 82 participants in each group, totaling at least 164 study participants.

### Statistical Methods

Statistical analyses were performed using SPSS (version 21.0; IBM Corp). Categorical variables are presented as frequencies and percentages and were compared between groups using the chi-square test. Continuous variables were assessed for normality using the Shapiro-Wilk test. Normally distributed data are expressed as means and SDs and were compared using the independent-sample 2-tailed *t* tests, whereas nonnormally distributed data are presented as medians and IQRs and were compared using the Mann-Whitney *U* test. For the comparison of postoperative LARS scores between groups, the Mann-Whitney *U* test was used because both baseline and postoperative LARS scores were not normally distributed (Shapiro-Wilk test; *P*<.001). For the comparison of LARS severity categories (no LARS, minor, or major), a multivariable ordinal logistic regression model was applied with adjustment for baseline LARS scores. The proportional odds assumption was assessed using the parallel lines test. A 2-sided *P* value of less than .05 was considered statistically significant.

### Ethical Considerations

This study was reviewed and formally approved by the ethics committee of the First Hospital of Jilin University (21K028-001). Written informed consent was obtained from all individual participants included in this study. All participant data were anonymized and handled confidentially in accordance with institutional and ethical guidelines. No financial compensation was provided to participants for their involvement in this study.

## Results

### Comparison of General Data Between the Two Groups of Patients

**Figure 4.**
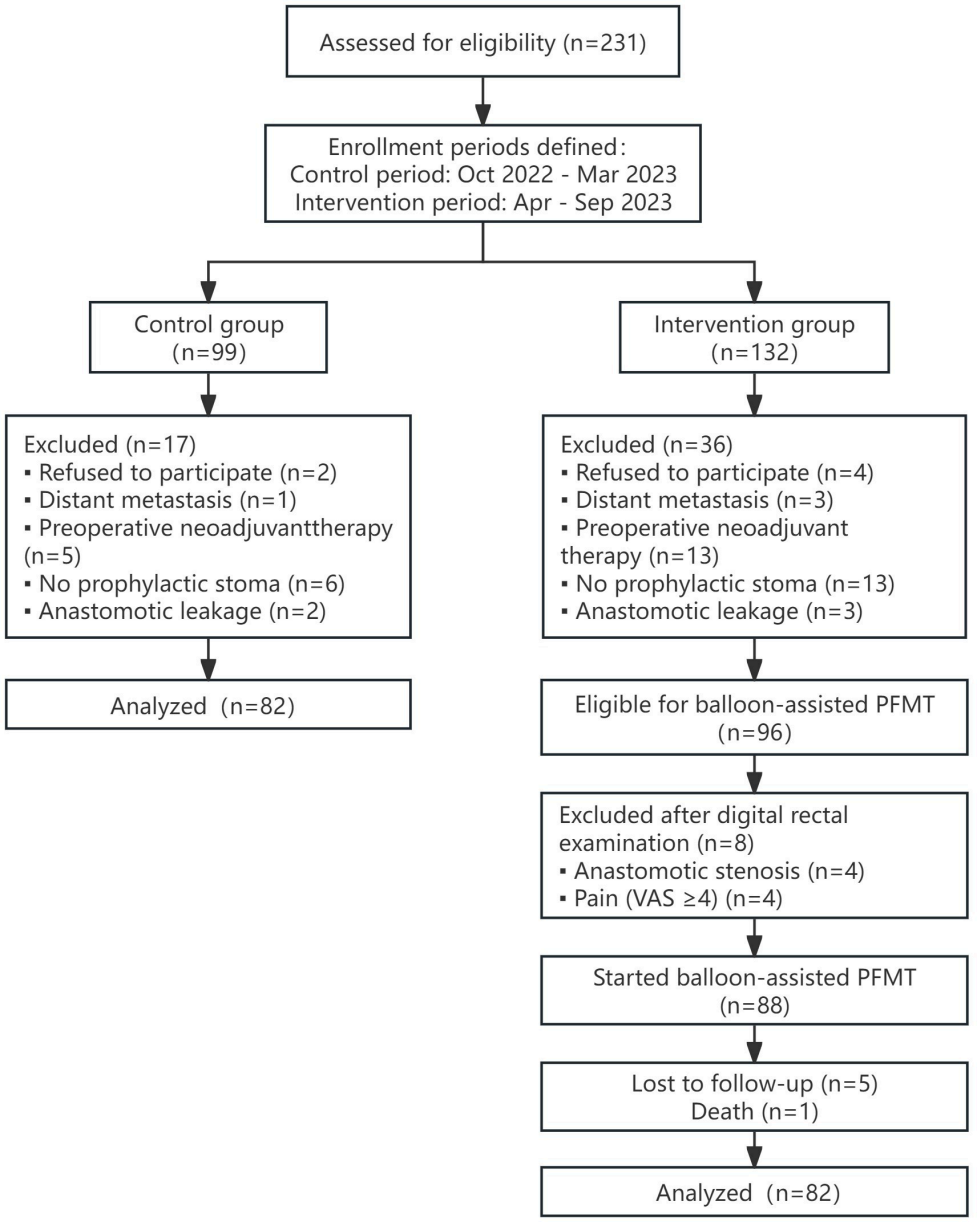
Flowchart of patient enrollment, exclusion, and allocation. PFMT: pelvic floor muscle training; VAS: visual analog scale.

From October 2022 to September 2023, a total of 231 patients with low rectal cancer were recruited and assessed for eligibility in this study. The participant enrollment process is detailed in Figure 4. In the control group, a total of 99 patients were screened, with 17 (17.2%) excluded, whereas in the intervention group, of the 132 patients screened, 36 (27.3%) were excluded based on baseline eligibility criteria. Of the remaining 96 patients eligible for balloon-assisted PFMT in the intervention group, 8 (8.3%) did not meet the postoperative safety criteria prior to intervention initiation. During follow-up, of the remaining 88 patients, 5 (5.7%) were lost to follow-up, and 1 (1.1%) died. Ultimately, 82 patients were included in the final analysis in the intervention group. Overall, 164 eligible patients were included in the study, with 82 (50%) in the intervention group and 82 (50%) in the control group. The 2 groups demonstrated comparable baseline characteristics, with no significant differences in demographic or clinical data (*P*>.05 in all cases). Additionally, baseline LARS scores prior to temporary stoma surgery showed no statistically significant intergroup discrepancy (*P*=.09). Detailed baseline data are shown in Table 1.

**Table 1.** Comparison of the general information of patients in the 2 groups.

Variables	Control group (n=82)	Intervention group (n=82)	*t* test (*df*), *Z* score, or chi-square (*df*)	*P* value
Age (y), mean (SD)	58.43 (9.34)	58.82 (10.64)	*t*_162_=−0.25	.80
Sex, n (%)			χ²_1_=0.1	.87
Male	56 (68.3)	58 (70.7)		
Female	26 (31.7)	24 (29.3)		
Pathological stage, n (%)			χ²_2_=0.8	.68
1	25 (30.5)	29 (35.4)		
2	29 (35.4)	24 (29.3)		
3	28 (34.1)	29 (35.4)		
Anastomotic technique, n (%)			χ²_1_=0.5	.68
Handsewn anastomosis	13 (15.9)	16 (19.5)		
Stapled anastomosis	69 (84.1)	66 (80.5)		
Place of residence, n (%)			χ²_1_=0.1	.87
Urban	51 (62.2)	49 (59.8)		
Rural	31 (37.8)	33 (40.2)		
Educational level, n (%)			χ²_2_=0.7	.71
Primary school or lower	34 (41.5)	30 (36.6)		
Junior high school, high school, or secondary school	39 (47.6)	40 (48.8)		
College or higher	9 (11.0)	12 (14.6)		
LARS^a^ score (0-42), median (IQR)	29.96 (27.00-39.00)	27.35 (22.25-34.00)	Z=−1.7	.09
LARS degree rating, n (%)			χ²_2_=3.6	.17
No	14 (17.1)	20 (24.4)		
Mild	17 (20.7)	23 (28.0)		
Severe	51 (62.2)	39 (47.6)		

a LARS: low anterior resection syndrome.

### Adverse Events

No device-related adverse events were reported in either group throughout the study period. Specifically, no patients experienced anal bleeding, severe pain (visual analog scale score of ≥4), mucosal injury, or infection associated with PFMT. All patients in both groups completed the training protocol without premature discontinuation due to adverse effects.

### Comparison of PFMT Accuracy of Technique, Adherence, and LARS Scores Between the Two Groups of Patients

Patients in the intervention group demonstrated significantly superior training accuracy and adherence compared to the control group (*P*<.001 in both cases; Table 2). Postoperative LARS scores were significantly lower in the intervention group than in the control group as assessed using the Mann-Whitney *U* test (*Z*=−3.491; *P*<.001). Furthermore, the distribution of LARS severity differed significantly between the 2 groups (χ²_2_=20.8; *P*<.001), with a higher proportion of patients without LARS and a lower proportion of severe LARS observed in the intervention group (Table 2). In addition, a multivariable-adjusted ordinal logistic regression model (accounting for baseline LARS scores) revealed that the intervention group had a markedly reduced risk of developing major LARS (odds ratio 0.27, 95% CI 0.15‐0.50; *P*<.001), as detailed in Table 3.

**Table 2.** Comparison of pelvic floor muscle training and low anterior resection syndrome (LARS) scores at 1 month after stoma reversal surgery between the 2 groups of patients.

Variables	Control group (n=82)	Intervention group (n=82)	*Z* score or chi-square (*df*)	*P* value
Accuracy of technique (%), median (IQR)	68.00 (56.00-80.25)	84.22 (73.00-98.50)	Z=−5.830	<.001
Adherence (%), median (IQR)	65.08 (50.00-84.17)	87.68 (83.33-100.00)	Z=−6.200	<.001
LARS score (0-42), median (IQR)	32.00 (13.75-36.00)	16.00 (11.00-29.00)	Z=−3.491	<.001
LARS degree rating, n (%)			χ²_2_=20.8	<.001
No	23 (28.0)	45 (54.9)		
Mild	14 (17.1)	20 (24.4)		
Severe	45 (54.9)	17 (20.7)		

**Table 3.** Multivariable ordinal logistic regression analysis of factors associated with low anterior resection syndrome (LARS) severity at 1 month after stoma reversal surgery. The model passed the parallel lines test (*P*=.40), satisfying the proportional odds assumption.

Variable	β (SE)	OR^a^ (95% CI)	*P* value
Group (intervention)	−1.310 (0.313)	0.27 (0.15‐0.50)	<.001
Baseline LARS score	0.051 (0.014)	1.05 (1.02‐1.08)	<.001

a OR: odds ratio.

### Comparison of Quality of Life Scores Between the Two Groups of Patients

At 1 month after temporary ileostomy reversal surgery, patients in the intervention group had significantly higher scores in physical functioning, role functioning, social functioning, and overall quality of life than the control group. Scores for insomnia, constipation, and diarrhea were significantly lower in the intervention group than in the control group, and the score on the economic difficulties dimension in the intervention group was notably lower than in the control group, as shown in Table 4.

**Table 4.** Comparison of European Organisation for Research and Treatment of Cancer Quality of Life Questionnaire Core 30 scores between the control and intervention groups at 1 month after stoma reversal surgery^a^.

Variable (0-100)	Control group (n=82), median (IQR)	Intervention group (n=82), median (IQR)	*Z* score	*P* value
Physical functioning	80 (73.33-86.67)	93.33 (86.67-100)	−6.303	<.001
Role functioning	66.67 (66.67-83.33)	83.33 (66.67-100)	−2.234	.03
Emotional functioning	91.67 (89.58-91.67)	91.67 (83.33-100)	−1.694	.09
Cognitive functioning	100 (100-100)	100 (100-100)	−1.821	.07
Social functioning	66.67 (66.67-66.67)	66.67 (66.67-100)	−2.948	.003
Global health and quality of life	75 (66.67-83.33)	83.33 (75-83.33)	−4.551	<.001
Fatigue	0 (0-11.11)	0 (0-0)	−1.924	.05
Nausea or vomiting	0 (0-0)	0 (0-0)	−1.743	.08
Pain	0 (0-0)	0 (0-0)	−0.672	.50
Dyspnea	0 (0-0)	0 (0-0)	−0.342	.73
Insomnia	0 (0-33.33)	0 (0-0)	−3.469	.001
Appetite loss	0 (0-0)	0 (0-0)	−1.108	.27
Constipation	0 (0-0)	0 (0-0)	−2.019	.04
Diarrhea	0 (0-33.33)	0 (0-0)	−3.308	.002
Financial difficulties	33.33 (33.33-33.33)	0 (0-33.33)	−4.694	<.001

a If both the 25th and 75th percentiles are 0, it indicates that at least 75% of the patients scored 0 on that item.

## Discussion

### Principal Findings

This study aimed to investigate the impact of a novel home-based integrated device for pelvic floor intra-anal balloon training and reminders on postoperative LARS in patients with low rectal cancer. The results showed that using this device to guide and monitor patients in home-based PFMT significantly improved the accuracy of technique and adherence to patient training, thereby reducing the incidence and severity of LARS at 1 month after temporary ileostomy reversal surgery and improving patients’ quality of life.

PFMT involves consciously and autonomously contracting the levator ani and anal sphincter muscles to increase the stiffness of the pelvic floor muscle group and reduce anal descent. This approach helps improve bowel control by enhancing voluntary sphincter contraction, which may indirectly improve rectal reservoir function and is beneficial in repairing injuries to the internal and external anal sphincters and perianal nerves following surgery [9,21]. PFMT is simple to learn and not limited by time or location and does not increase patient discomfort. Several studies have demonstrated the effectiveness of PFMT in improving symptoms of LARS in patients after rectal cancer surgery [22].

Intra-anal balloon training, as an auxiliary method for PFMT, can help patients better perceive and exercise the pelvic floor muscles, providing real-time feedback that allows patients to perform muscle contractions and relaxations more accurately, thus achieving better exercise results. However, research on the use of intra-anal balloon training in PFMT is still relatively limited [23], and there is currently a lack of commercially available balloon devices that can be widely used.

In this study, the design and use of the intra-anal balloon training and reminder integrated device provided an innovative approach to the rehabilitation of patients with low rectal cancer. By combining an adjustable-pressure balloon device with a dynamic monitoring device, patients can perform PFMT at home and ensure the accuracy of technique and adherence to the training through real-time monitoring and feedback. In this study, comprehensive and rich health education content related to LARS prevention for patients with mid-low rectal cancer was provided, and postoperative daily follow-ups and PFMT were widely and meticulously promoted. The results showed that adherence was higher in the intervention group than in the control group (median 87.68%, IQR 83.33%-100.00% vs 65.08%, IQR 50.00%-84.17%; *P*<.001) and much higher than previously reported rates [12]. After adjusting for baseline LARS scores, the intervention was associated with a 73% reduction in the risk of major LARS (odds ratio 0.27, 95% CI 0.15‐0.50), suggesting that the device has significant clinical implications for improving postoperative bowel function.

In summary, the results of this study suggest that the self-designed intra-anal balloon training and reminder integrated device enables self-management of home-based PFMT and has potential clinical applications in preventing and improving LARS in patients with low rectal cancer, warranting widespread clinical promotion.

### Limitations

This study is the first nonrandomized controlled trial integrating intra-anal balloon training with a reminder device for the prevention of LARS. To minimize group contamination caused by cross-contact between concurrent patients, a temporal isolation strategy was adopted. In addition, the interventions were standardized through systematic training by the same nursing team. Nevertheless, several potential biases should be considered. First, the nonrandomized, time-sequenced design may introduce unmeasured time-related confounding (eg, potential differences in surgical techniques or perioperative care over the recruitment period). In addition, the exclusion rate differed between the 2 recruitment periods, which may have contributed to baseline differences in functional status between groups. Second, although the analysis of baseline characteristics and intensive follow-up management in the control group suggested comparability between groups, the biofeedback devices used in the intervention group may introduce a slight Hawthorne effect bias by objectively quantifying training parameters. Third, training adherence in both groups was manually recorded via self-reported electronic diaries, which may have introduced self-reporting bias. The combination of this self-reporting bias and the Hawthorne effect from intensive follow-up and device feedback may have led to overestimation of adherence in the intervention group, potentially exaggerating the observed differences between groups. Fourth, this study did not perform a strict intention-to-treat analysis as participants who were lost to follow-up were excluded from the final analysis. Although the overall attrition rate was low, this may introduce potential attrition bias and should be considered when interpreting the results.

Additionally, the single-center sample source and the relatively short follow-up period represent important limitations of this study. Outcomes were assessed only 1 month after temporary ileostomy reversal, which mainly reflects early postoperative bowel function recovery rather than long-term functional outcomes. Previous randomized controlled trials have reported inconsistent evidence regarding the long-term effectiveness of PFMT for LARS, with some studies showing only transient benefits over time. Therefore, the findings of this study should primarily be interpreted as indicating a potential benefit in early functional recovery rather than definitive long-term improvement. Furthermore, given that neoadjuvant therapy (particularly radiotherapy) may lead to anastomotic tissue fibrosis, reduced blood supply, delayed healing, and potential complications (eg, bleeding or dehiscence) when initiating intra-anal balloon training 1 month after stoma reversal, we prioritized validating the device’s efficacy in low-risk patients who did not receive neoadjuvant radiotherapy to ensure intervention safety, and therefore, the results cannot be generalized to higher-risk patient groups. Future phase 2 studies will expand to high-risk populations undergoing neoadjuvant therapy. To address these limitations, we plan to conduct a multicenter stepped-wedge cluster randomized controlled trial that includes patients receiving neoadjuvant therapy, analyzes the association between radiotherapy dose and type and LARS, and further evaluates the device’s clinical utility.

## Abbreviations

DRE: digital rectal examination
LARS: low anterior resection syndrome
PFMT: pelvic floor muscle training
